# Efficacy and Safety of 6.3 Fr Versus 7.5 Fr Single-Use Flexible Ureteroscopes for Upper Urinary Tract Stones: A Systematic Review and Meta-Analysis of Randomized Controlled Trials

**DOI:** 10.3390/medicina61122103

**Published:** 2025-11-26

**Authors:** Abdullah M. Alharran, Saad A. Alajmi, Ali A. Hussain, Mohammad Salem Alajmi, Sayed Hashim, Layal T. Alwazzan, Husain Alaradi

**Affiliations:** 1 Faculty of Medicine, Arabian Gulf University, Manama 329, Bahrain; 2 Kuwait Institute for Medical Specializations, Kuwait City 12050, Kuwaithashem2592@moh.gov.kw (S.H.); 3 Department of Surgery, Al Amiri Hospital, Kuwait City 12050, Kuwait; hussain5100@moh.gov.kw; 4 Faculty of Medicine, Jordan University of Science and Technology, Irbid 22110, Jordan; msajami17@med.just.edu.jo; 5 Royal College of Surgeons in Ireland, Alsayah 229, Bahrain; 24206960@rcsi.com; 6 Department of Urology, Arabian Gulf University, Manama 329, Bahrain; haradi8@health.gov.bh

**Keywords:** kidney stone, urology, urolithiasis, ureteroscopy, retrograde intrarenal surgery

## Abstract

*Background and Objectives*: Retrograde intrarenal surgery (RIRS) is a cornerstone in managing upper urinary tract stones, with a growing trend towards instrument miniaturization. The introduction of the ultra-slim 6.3 Fr single-use flexible ureteroscope presents a potential advancement over the standard 7.5 Fr device, but clinical evidence remains scarce. This systematic review and meta-analysis aims to synthesize data from randomized controlled trials (RCTs) to compare the efficacy and safety of the 6.3 Fr versus the 7.5 Fr ureteroscope. *Materials and Methods*: A systematic search of PubMed, Scopus, CENTRAL, and Web of Science was conducted for RCTs published up to September 2025. The primary outcomes were the stone-free rate and procedural success rate. Secondary outcomes included operation duration and postoperative complications. Risk ratios (RRs) for dichotomous data and mean differences (MDs) for continuous data, with 95% confidence intervals (CIs), were pooled for analysis. *Results*: Three RCTs involving 140 patients were included. There was no significant difference between both groups in stone-free rates (RR: 1.06, 95% CI [0.96, 1.18], *p* = 0.22) or success rates (RR: 1.06, 95% CI [0.97, 1.16], *p* = 0.17). However, the 6.3 Fr ureteroscope was associated with a significantly shorter operation duration (MD: −6.66 min, 95% CI [−11.29, −2.03], *p* < 0.001). No significant differences were found in laser operating time (MD: −1.46 min, 95% CI [−3.93, 1.01], *p* = 0.25), length of hospital stay (MD: −0.09 days, 95% CI [−0.23, 0.05], *p* = 0.19), or postoperative complications (Clavien I: RR 0.86, 95% CI [0.30, 2.43], *p* = 0.77; Clavien II–III: RR 0.67, 95% CI [0.12, 3.84], *p* = 0.65). *Conclusions*: Based on low-certainty evidence, the 6.3 Fr ureteroscope does not significantly improve stone-free rates but may reduce overall operation duration compared to the 7.5 Fr scope, with a comparable safety profile. These findings are limited by the small number of available studies, highlighting a clear need for larger, high-quality RCTs to confirm these preliminary results.

## 1. Introduction

Retrograde intrarenal surgery (RIRS) is the cornerstone of minimally invasive management for upper urinary tract stones [1]. Since the first fiber-optic ureteroscope was developed in the late 1980s [2], the field has undergone significant technological advancements. Digital, disposable devices have increasingly replaced older fiber-optic instruments, though reusable scopes remain widely utilized depending on economic and institutional factors [3]. This key update in procedure has been facilitated by multiple benefits, including the eradication of cross-contamination risk, the elimination of expensive maintenance and sterilization expenses, and the assurance of stable, top-tier performance with every new device [4]. This progress sets the stage for today’s cutting-edge technology and the persistent pursuit of improvement.

A rising trend in endourology is the gradual reduction in size of surgical instruments [5]. This trend is clinically justified because smaller ureteroscopes are meant to decrease ureteral injury and improve navigation through constricted or unstented ureters, and may help to reduce postoperative complications [6]. Despite these significant benefits, their extent can be influenced by several factors such as surgeon experience [7]. Additionally, while usage trends may vary by institution and region, the 7.5 Fr flexible ureteroscope remains a widely used standard, offering reliable performance in a smaller size. Still, introducing the ultra-slim 6.3 Fr single-use flexible ureteroscope represents the latest advancement [8]. This development presents a crucial clinical question: Does this considerable reduction in diameter lead to observable improvements in patient outcomes, while maintaining essential aspects, such as image quality, deflection, and long-term reliability?

Although some early randomized controlled trials (RCTs) comparing the new 6.3 Fr scope to the standard 7.5 Fr model have been released, the data are still scarce, warranting further evidence synthesis to guide clinical practice [9,10,11]. Therefore, the primary objective of this study is to conduct the first systematic review and meta-analysis of RCTs to rigorously compare the 6.3 Fr and 7.5 Fr single-use flexible ureteroscopes. We will assess efficacy, primarily measured by stone-free rates, and safety, measured by postoperative complication rates.

## 2. Materials and Methods

### 2.1. Protocol Registration

Registration of this systematic review was pre-completed through the (CRD420251152225) in the International Prospective Register of Systematic Reviews (PROSPERO). The procedures for this systematic review and meta-analysis complied with the PRISMA guidelines [12] and the Cochrane Handbook for Systematic Reviews of Interventions [13].

### 2.2. Data Sources & Search Strategy

On 16 September 2025, a literature search was systematically conducted by (A. M. H.) across the following electronic databases: PubMed, Scopus, CENTRAL, and Web of Science. The search strategy utilized a string of the following keywords: (ureteroscopy OR ureteroscop* OR RIRS OR “retrograde intrarenal surgery” OR “flexible ureteroscope*”) AND (“6.3 Fr” OR “6.3 Fr” OR “7.5 Fr” OR “7.5 Fr” OR “ultra-slim” OR “ultraslim” OR “small diameter”). The search terms and database results are comprehensively summarized in (Table S1). No language restrictions or other search filters were used. We also manually reviewed the reference sections of eligible trials to ensure comprehensive coverage and to avoid overlooking any relevant studies.

### 2.3. Eligibility Criteria

RCTs were included if they followed the following Population, Intervention, Control, and Outcome (PICO) framework:

1. Population (P): adult patients with upper urinary tract stones (kidney or proximal ureteral) of small-to-moderate size (≤2 cm) who are candidates for flexible ureteroscopy.
2. Intervention (I): RIRS performed using a 6.3 Fr single-use flexible ureteroscope.
3. Control (C): RIRS performed using a 7.5 Fr single-use flexible ureteroscope.
4. Outcomes (O): The primary outcome was the stone-free rate (as defined by the individual trial protocols) and success rate, defined as the successful completion of the planned stone clearance, including the ability to advance the ureteroscope to the target stone and not converting to open surgery. Secondary outcomes included operation duration, laser operating time, length of hospital stay (LoS), and Clavien complications.

The following criteria were used to exclude studies: quasi-randomized studies, conference abstracts or proceedings, study protocols, and observational studies or reviews.

### 2.4. Study Selection

Using the Covidence online platform, two reviewers (S.A.A. and A.A.H.) independently assessed the eligibility of the retrieved records. Once the duplicates were removed automatically, the remaining unique articles were screened in two stages. We screened titles and abstracts, then assessed the full text of potentially eligible studies. Conflicts between reviewers were resolved through discussion, resulting in a consensus.

### 2.5. Data Extraction

The data extraction was conducted independently by two reviewers (M. S. A. and S. H.). Discrepancies were resolved through discussion and in consultation with a senior author. The data extraction process involved creating an Excel spreadsheet, which was tested in a pilot phase before the final extraction. The extraction form was categorized into three primary sections:

Study characteristics: study ID, country, study design, total number of patients, intervention group details, control group details, main inclusion criteria, primary outcome, and follow-up duration.

1. Participant baseline characteristics: age, body mass index (BMI), gender, stone size, stone volume, stone number, hydronephrosis, and pre-procedural stenting.
2. Outcome data: stone-free rate, success rate, operation duration, laser operating time, LoS, and Clavien complications.

### 2.6. Risk of Bias and Certainty of Evidence

The revised Cochrane Collaboration’s Risk of Bias tool (ROB 2) [14] was employed to assess the methodological quality and bias risk for each RCT. Two reviewers (S. H. and L. T. A) independently evaluated each study across domains such as selection bias, performance bias, reporting bias, and attrition bias. Disagreements were resolved by consensus. Additionally, the overall certainty of the evidence was assessed using the Grading of Recommendations Assessment, Development, and Evaluation (GRADE) approach [15,16]. This framework considers risk of bias, inconsistency, indirectness, imprecision, and publication bias. Each factor was carefully assessed, and the rationale for each judgment was documented, with any discrepancies resolved through discussion.

### 2.7. Statistical Analysis

Statistical analyses were conducted using Stata MP version 17 (Stata Corp., College Station, TX, USA). The risk ratio (RR) was computed for dichotomous outcomes, and the mean difference (MD) was calculated for continuous outcomes, both presented alongside the corresponding 95% confidence intervals (CI). Heterogeneity was evaluated using the chi-squared (χ^2^) test and the I^2^ statistic. A *p*-value less than 0.1 for the χ^2^ test or an I^2^ value of 50% or higher indicated significant heterogeneity. The default analysis model was fixed-effects due to the limited number of included trials, which limits the precision of between-study variance estimation in random-effects models. A random-effects model was applied only if significant heterogeneity was observed, defined as a Chi-squared *p* < 0.1 or an I^2^ > 50%. An assessment for publication bias was not performed, as all analyzed outcomes included fewer than 10 RCTs [17].

## 3. Results

### 3.1. Search Results and Study Selection

**Figure 1:**
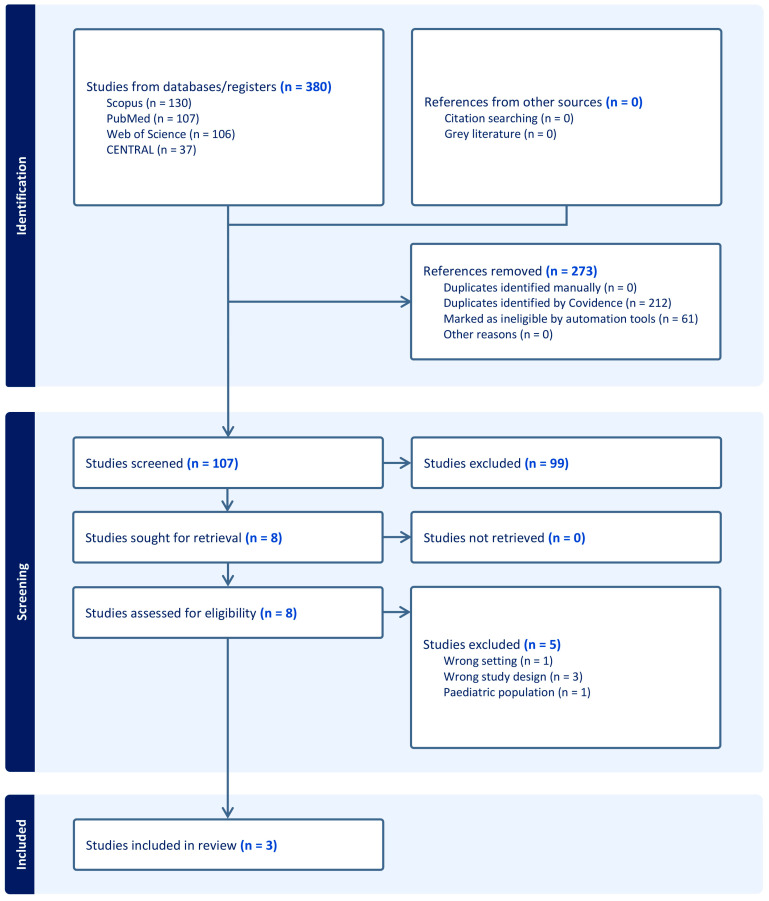
PRISMA flow chart of the screening process.

A total of 380 records were retrieved from the initial literature search. Following the automated exclusion of 273 irrelevant records, the titles and abstracts of the remaining 107 articles underwent screening. Consequently, 99 studies were excluded for failing to satisfy the inclusion criteria. Thus, eight articles were evaluated for eligibility by screening their full texts. Five studies were excluded for various reasons (Table S2). Ultimately, three RCTs [9,10,11] were included in the qualitative and quantitative synthesis (Figure 1).

### 3.2. Characteristics of Included Studies

This review included three RCTs, with a total of 140 patients [9,10,11]. The included population consisted of adult patients with small-to-moderate upper urinary tract stones, with maximum size criteria ranging from <1.5 cm to ≤2 cm. Some differences in the procedure methodology were noted in the use of a ureteral access sheath (UAS): one trial used a sheathless technique for the intervention group [9], another used a suction UAS for all subjects [11], and the third utilized a standard UAS selectively in 47% of the cases [10]. Further key details about the study designs are summarized in (Table 1). Also, information about the included patients’ baseline data is summarized in (Table 2).

### 3.3. Risk of Bias and Certainty of Evidence

**Figure 2:**
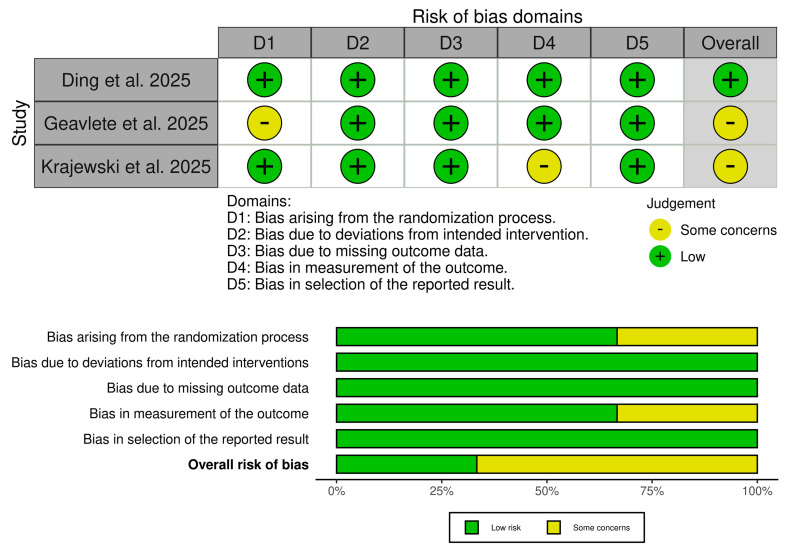
Quality assessment of risk of bias in the included trials. The upper panel presents a schematic representation of risks (low = green, unclear = yellow) for specific types of biases of the studies in the review. The lower panel presents risks (low = green, unclear = yellow, and high = red) for the subtypes of biases of the combination of studies included in this review [9, 10, 11].

Ding et al. showed an overall low risk of bias [9], while the other two trials showed some concerns [10,11] (Figure 2). Geavelte et al. showed some concerns about selection bias due to the absence of details of the randomization procedure [11]. Krajewski et al. showed some concerns of detection bias as the stone-free rate was assessed intraoperatively and subjectively by the unblinded operating surgeon [10]. Additionally, the certainty of evidence assessment is outlined in (Table 3).

### 3.4. Primary Outcomes

**Figure 3:**
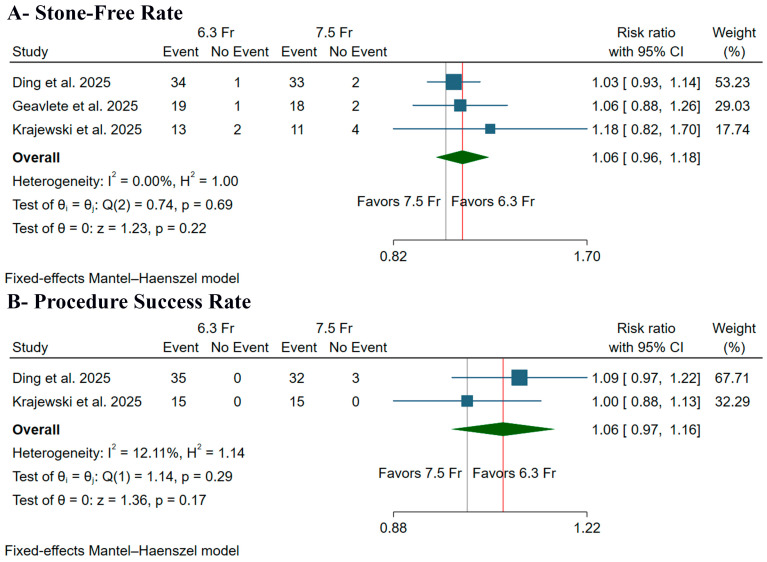
Forest plots of the primary outcomes, CI: confidence interval [9,10,11].

There was no significant difference between the two ureteroscope sizes regarding the incidence of stone-free rate, which was assessed either intraoperatively or at one-month post-surgery, (RR: 1.06, 95% CI [0.96, 1.18], *p* = 0.22) (Figure 3A) or procedure success rate (RR: 1.06, 95% CI [0.97, 1.16], *p* = 0.17) (Figure 3B). Pooled studies were homogeneous in both stone-free rate (I^2^ = 0%) and procedure success rate (I^2^ = 12.11%).

### 3.5. Secondary Outcomes

#### 3.5.1. Secondary Procedural Outcomes

**Figure 4:**
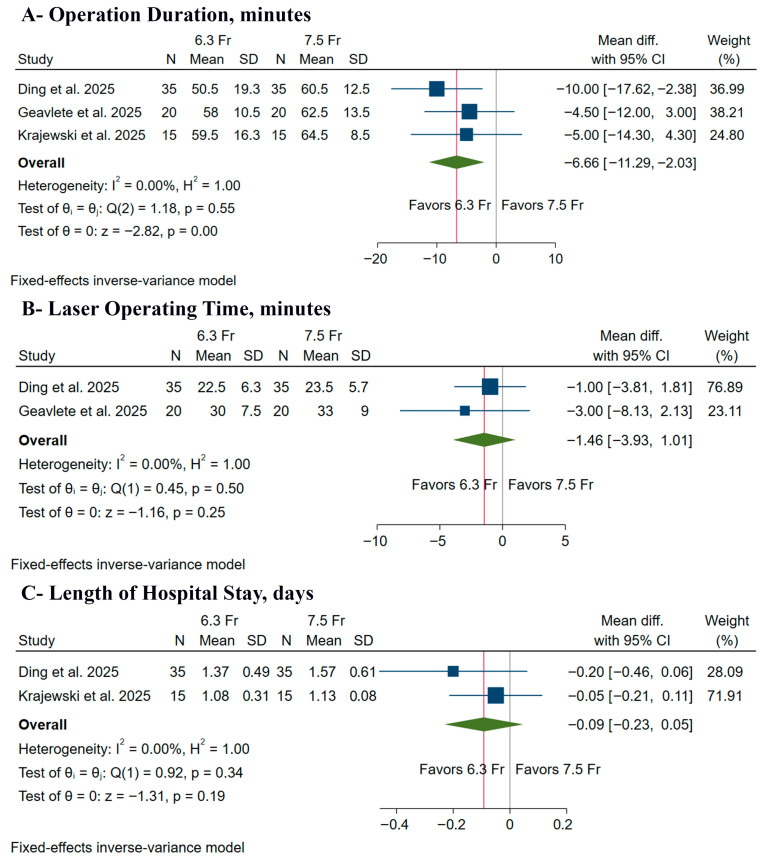
Forest plots of the secondary procedural outcomes, CI: confidence interval [9,10,11].

The 6.3 Fr ureteroscope was associated with a significantly shorter operation duration (MD: −6.66 min, 95% CI [−11.29, −2.03], *p* < 0.001) (Figure 4A); however, there was no significant difference between both groups in laser operating time (MD: −1.46 min, 95% CI [−3.93, 1.01], *p* = 0.25) (Figure 4B) or LoS (MD: −0.09 days, 95% CI [−0.23, 0.05], *p* = 0.19) (Figure 4C). Pooled studies were homogeneous in operation duration (I^2^ = 0%), laser operating time (I^2^ = 0%), and LoS (I^2^ = 0%).

#### 3.5.2. Secondary Safety Outcomes

**Figure 5:**
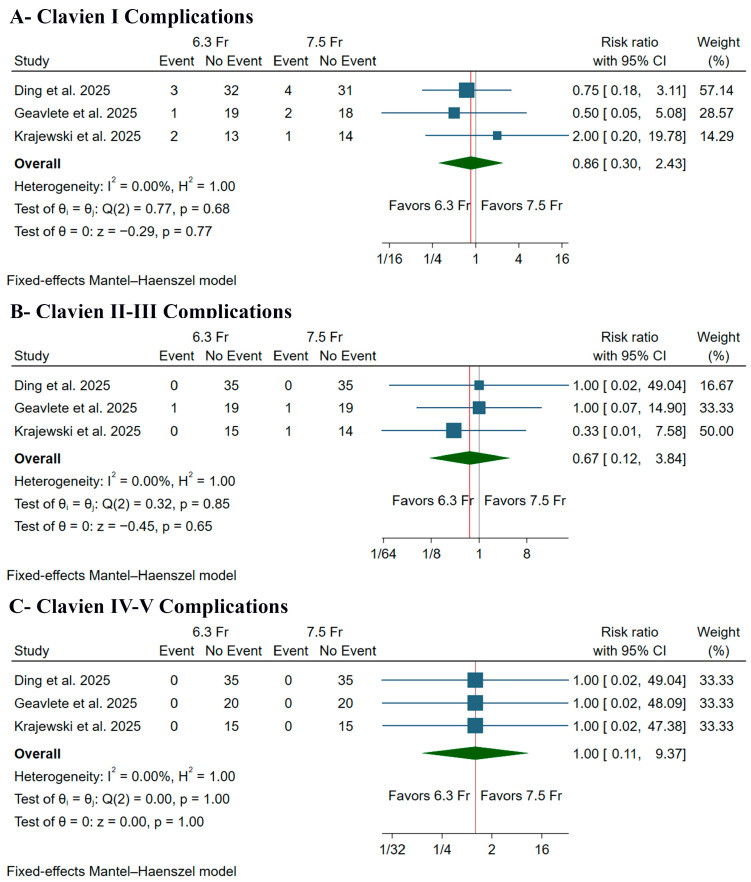
Forest plots of the safety outcomes, CI: confidence interval [9,10,11].

There was no significant difference between the two strategies regarding the incidence of Clavien I complications (RR: 0.86, 95% CI [0.30, 2.43], *p* = 0.77) (Figure 5A), Clavien II–III complications (RR: 0.67, 95% CI [0.12, 3.84], *p* = 0.65) (Figure 5B), or Clavien IV–V complications (RR: 1.00, 95% CI [0.11, 9.37], *p* = 1.00) (Figure 5C). Pooled studies were homogeneous in Clavien I complications (I^2^ = 0%), Clavien II–III complications (I^2^ = 0%), and Clavien IV–V complications (I^2^ = 0%).

## 4. Discussion

After synthesizing three RCTs and 140 patients, there was no difference in 6.3 Fr and 7.5 Fr ureteroscope sizes in terms of stone-free rate, procedural success rate, laser operating time, LoS, and complications. Still, patients treated with a 6.3 Fr ureteroscope showed a significantly shorter operation duration compared to those treated with a 7.5 Fr ureteroscope. Additionally, the evidence remains uncertain due to the limited number of trials, underscoring the need for further investigation.

In current clinical practice, the selection of ureteroscope sizes often depends on the surgeon’s experience, patient-specific ureteral conditions, and stone characteristics [7]. However, robust evidence-based guidance to inform this decision is lacking, which highlights the need for a comprehensive evidence synthesis. Accordingly, our primary efficacy outcome, the stone-free rate, showed no statistically significant difference between the two ureteroscopes. This result is difficult to interpret, mainly because the studies used different ways to define and measure SFR, which our GRADE assessment considered a “serious indirectness”. Stone-free status was evaluated in two trials using a postoperative non-contrast computed tomography scan, which is the most reliable method [9,11]. Conversely, one study utilized a subjective, unblinded intraoperative visual evaluation conducted by the operating surgeon, which is highly susceptible to detection bias, as numerous studies have demonstrated that surgeons’ intraoperative estimates of stone clearance are often overly optimistic and correlate poorly with postoperative imaging [18,19].

Despite this critical variance in stone-free rate evaluation, statistical heterogeneity was not observed, likely reflecting the meta-analysis’s low statistical power to detect heterogeneity with only three small studies, rather than a truly consistent treatment effect [20]. Still, the observed positive trend in SFR with the 6.3 Fr scope could theoretically be attributed to improved irrigation dynamics, which was supported by previous preclinical studies [21,22,23]. When used with a UAS, the smaller scope results in a larger sheath-to-scope ratio. This may improve irrigation outflow, lower intrarenal pressure, and potentially facilitate more efficient passive evacuation of stone dust and small fragments, contributing to improved overall clearance [21,22,23].

Moreover, the 6.3 Fr ureteroscope showed a statistically significant advantage, reducing the mean operation duration by 6.66 min, though the evidence supporting this is of low certainty. This effect was primarily driven by Ding et al., who had the greatest impact on the pooled analysis. They utilized a “sheathless” RIRS for the 6.3 Fr group, while the 7.5 Fr group was treated with a conventional UAS approach. The reduction in operation time in the 6.3 Fr arm can be attributed to the complete elimination of the UAS placement step. This step was time-consuming and caused three patients in the 7.5 Fr cohort to experience access failure, thereby extending the procedure duration [9]. However, other potential confounders, such as surgeon experience [7], stone density [11], and preoperative stenting [24] may also influence procedural duration and were not uniformly controlled across all trials.

Beyond the sheathless approach, the availability of the 6.3 Fr ureteroscope opens new possibilities for sheath selection. Although the included studies utilized standard 10/12 Fr sheaths, the ultra-slim scope can theoretically permit the use of smaller access sheaths (9.5/11.5 Fr). While not tested in the current analysis, future research could investigate if this combination mitigates the risk of ureteral injury [10,11], as this method may reduce the risk of ureteral injury during sheath insertion, offering a substantial advantage for patients with narrow ureters [25]. However, this strategy presents a trade-off, as employing a smaller sheath could diminish the gap between the scope and the sheath, which might impede irrigation outflow and obstruct the passive elimination of stone fragments, potentially compromising the effectiveness of stone clearance [26]. Geavlete et al. highlighted this notion; using the 6.3 Fr scope within a standard 10/12 Fr sheath created a more favorable scope-to-sheath ratio (63%) compared to a 7.5 Fr scope (75%), thereby improving the available working space for irrigation and fragment removal [11].

Accordingly, the reduced diameter of the ultra-slim scope offers a notable operational benefit, allowing for a more practical sheathless approach, especially in cases of unstented or narrowed ureters, which subsequently facilitates the procedure and reduces the operation duration [27]. Still, the non-significant findings for laser operating time and LoS are justified. Laser time is primarily dependent on stone characteristics, such as volume and density, rather than scope diameter [11]. At the same time, LoS for uncomplicated RIRS is already minimal, leaving little room for a clinically meaningful or measurable difference between the groups [9,10].

Furthermore, the safety profiles of both ureteroscopes were excellent, with no severe complications (Clavien-Dindo grade IV–V) reported in any of the 140 patients across the three trials. Still, our pooled analysis revealed a non-significant trend toward fewer minor complications with the 6.3 Fr scope, including Clavien-Dindo grade I (RR 0.86) and grades II–III (RR 0.67) events. This is consistent with the core clinical justification for using smaller instruments in endourology: ideally, a smaller instrument will place less mechanical strain on the ureteral wall, resulting in less injury to the lining, fewer complications after surgery, and a decreased risk of long-term issues, such as ureteral strictures [28,29]. However, the analysis lacked sufficient power to identify a real difference in safety results.

### Strengths & Limitations

To our knowledge, this meta-analysis is the first to address this specific clinical question. It was conducted according to rigorous PRISMA and Cochrane methodologies, including only RCTs to minimize selection bias, and providing a transparent, formal GRADE assessment of the certainty of the evidence for each outcome.

Despite these strengths, the conclusions are limited by a few limitations. First, there is a low number of included trials, with only three small trials and 140 patients. Hence, the analysis lacks the statistical power to provide precise effect estimates for most outcomes, particularly for rare events like complications. This imprecision is a primary reason for downgrading the certainty of evidence. Second, the analysis was challenged by clinical heterogeneity, which comprises the inconsistent use of a UAS, as previously discussed. To clarify, this issue is most prominent in the analysis of operation duration, where the pooled result was mainly driven by the Ding et al. study [9], which accounts for half of the patients in this meta-analysis. In that trial, the 6.3 Fr group underwent a sheathless procedure while the 7.5 Fr group did not, meaning the comparison was between two different surgical techniques, not just two instruments. Third, the variable definitions and assessment methods for the stone-free rate, as previously discussed, introduce serious methodological heterogeneity and a high risk of detection bias in one trial.

Fourth, another significant source of heterogeneity was the inconsistent use of preoperative stenting. The included trials reported different rates: 100% of patients were pre-stented in the study by Geavlete et al. [11], 56.7% in the Krajewski et al. trial [10], and none in the Ding et al. study [9], where pre-stenting was an exclusion criterion. Preoperative stenting is known to passively dilate the ureter, facilitating easier instrument access and potentially reducing procedural difficulty; this represents a critical confounding factor [24]. This difference probably affected some outcomes, such as procedural success, as the only instances of access failure noted in our analysis occurred in the non-stented 7.5 Fr cohort reported by Ding et al. [9]. Finally, the overall certainty of the evidence ranged from low to very low, reflecting the inherent limitations that warrant careful clinical interpretation of our results.

## 5. Conclusions

The 6.3 Fr ureteroscope did not significantly enhance clinical outcomes, including the primary outcome of stone-free rate, which was assessed at one month postoperatively or intraoperatively. Additionally, the 6.3 Fr ureteroscope significantly reduced the operation duration and demonstrated a favorable safety profile. However, these results are based on an overall low to very low certainty of evidence according to the GRADE assessment, reflecting the limited number of trials and their methodological inconsistencies. Therefore, current evidence is insufficient to recommend a routine shift to 6.3 Fr ureteroscopes in clinical practice. Further high-quality, multicenter RCTs are required to confirm these preliminary findings.

## Figures and Tables

**Table 1:** Summary characteristics of the included RCTs.

Study ID	Study Design	Country	Total Participants	Intervention Details	Control Details	Main Inclusion Criteria	Primary Outcome	Stone-Free Rate Definition and Measurement	Follow-Up Duration
Ding et al., 2025 [9]	RCT	China	70	Sheathless technique with a 6.3 Fr disposable ureteroscope and gravity irrigation.	A 10/12 Fr ureteral access sheath was used with a 7.5 Fr disposable ureteroscope and an irrigation pump.	Patients aged 18–75 with upper urinary tract stones < 1.5 cm in diameter.	Stone-free rate	No residual stones or asymptomatic fragments ≤ 2 mm; Non-contrast CT; 1-month follow-up	30 days
Geavlete et al., 2025 [11]	RCT	Romania	40	A 6.3 Fr single-use ureteroscope was used with a 10/12 Fr flexible suction ureteral access sheath in all cases.	A 7.5 Fr single-use ureteroscope was used with the same 10/12 Fr flexible suction ureteral access sheath in all cases.	Patients with kidney stones ≤ 2 cm who were pre-stented prior to surgery.	Stone-free rate	Absence of residual fragments larger than 2 mm; Non-contrast CT; 1-month	30 days
Krajewski et al., 2025 [10]	RCT	Poland	30	A 6.3 Fr digital disposable ureteroscope was used. A ureteral access sheath was utilized in 47% of all cases.	A 7.5 Fr digital disposable ureteroscope was used. A ureteral access sheath was utilized in 47% of all cases.	Adult patients with a single renal stone < 1.5 cm or multiple stones with a total volume < 850 mm^3^.	Stone-free rate	Absence of stone fragments larger than twice the laser fiber’s diameter; Intraoperative visual assessment; Intraoperatively	Early postoperative period (discharge within 24 h).

cm, centimeter; CT, computed tomography; Fr, French; h, hour; mm, millimeter; mm^3^, cubic millimeter; RCT, randomized controlled trial.

**Table 2:** Baseline characteristics of the participants.

Study ID	Number of Patients in Each Group		Age (Years), Mean (SD)		Gender (Female), N. (%)		Stone Size [mm], Mean (SD)		Stone Volume (mm^3^), Mean (SD)		Stone Number (Solitary/Multiple), N. (%)		Hydronephrosis, N. (%)		Preoperative Stenting, N. (%)	
	6.3 Fr	7.5 Fr	6.3 Fr	7.5 Fr	6.3 Fr	7.5 Fr	6.3 Fr	7.5 Fr	6.3 Fr	7.5 Fr	6.3 Fr	7.5 Fr	6.3 Fr	7.5 Fr	6.3 Fr	7.5 Fr
Ding et al., 2025 [9]	35	35	51.86 ± 12.89	52.77 ± 11.34	16 (45.71)	14 (40)	12.1 ± 2.3	12.9 ± 1.9	NR	NR	NR	NR	7 (20.0)	9 (25.7)	0 (0)	0 (0)
Geavlete et al., 2025 [11]	20	20	52.0 ± 12.5	49.0 ± 12.8	NR	NR	NR	NR	1370.5 ± 813.0	1247.0 ± 861.0	NR	NR	NR	NR	20 (100)	20 (100)
Krajewski et al., 2025 [10]	15	15	49.0 ± 22.2	51.0 ± 10.7	9 (60)	3 (20)	10.0 ± 3.7	9.0 ± 1.9	500.0 ± 543.7	364.5 ± 219.4	7 (46.7)/8 (53.3)	8 (53.3)/7 (46.7)	NR	NR	10 (66.7)	7 (46.7)

Fr, French; mm, millimeter; mm^3^, cubic millimeter; N, number of patients; NR, not reported; SD, standard deviation.

**Table 3:** GRADE evidence profile.

Certainty Assessment							Summary of Findings				
Participants (Studies) Follow-Up	Risk of Bias	Inconsistency	Indirectness	Imprecision	Publication Bias	Overall Certainty of Evidence	Study Event Rates (%)		Relative Effect (95% CI)	Anticipated Absolute Effects	
							With [7.5 Fr]	With [6.3 Fr]		Risk with [7.5 Fr]	Risk Difference with [6.3 Fr]
Stone-Free Rate											
140 (3 RCTs)	not serious	not serious	serious ^a^	serious ^b^	none	⨁⨁◯◯ Low ^a,b^	62/70 (88.6%)	66/70 (94.3%)	RR 1.06 (0.96 to 1.18)	62/70 (88.6%)	53 more per 1000 (from 35 fewer to 159 more)
Success Rate											
100 (2 RCTs)	not serious	not serious	not serious	extremely serious ^c^	none	⨁◯◯◯ Very low ^c^	47/50 (94.0%)	50/50 (100.0%)	RR 1.06 (0.97 to 1.16)	47/50 (94.0%)	56 more per 1000 (from 28 fewer to 150 more)
Operation Duration											
140 (3 RCTs)	not serious	not serious	not serious	very serious ^d^	none	⨁⨁◯◯ Low ^d^	-	-	-	-	MD 6.66 min lower (11.29 lower to 2.03 lower)
Laser Operating Time											
110 (2 RCTs)	not serious	not serious	not serious	extremely serious ^d^	none	⨁◯◯◯ Very low ^d^	-	-	-	-	MD 1.46 min lower (3.93 lower to 1.01 higher)
Length of Hospital Stay											
100 (2 RCTs)	not serious	not serious	not serious	extremely serious ^d^	none	⨁◯◯◯ Very low ^d^	-	-	-	-	MD 0.09 days lower (0.23 lower to 0.05 higher)
Clavien I Complications											
140 (3 RCTs)	not serious	not serious	not serious	very serious ^b^	none	⨁⨁◯◯ Low ^b^	7/70 (10.0%)	6/70 (8.6%)	RR 0.86 (0.30 to 2.43)	7/70 (10.0%)	14 fewer per 1000 (from 70 fewer to 143 more)
Clavien II–III Complications											
140 (3 RCTs)	not serious	not serious	not serious	very serious ^b^	none	⨁⨁◯◯ Low ^b^	2/70 (2.9%)	3/70 (4.3%)	RR 0.67 (0.12 to 3.84)	2/70 (2.9%)	9 fewer per 1000 (from 25 fewer to 81 more)
Clavien IV–V Complications											
140 (3 RCTs)	not serious	not serious	not serious	very serious ^b^	none	⨁⨁◯◯ Low ^b^	0/70 (0.0%)	0/70 (0.0%)	RR 1.00 (0.11 to 9.37)	0/70 (0.0%)	0 fewer per 1000 (from 0 fewer to 0 fewer)

CI: confidence interval; MD: mean difference; RR: risk ratio; Explanations: ^a^ The definition of the stone-free rate was heterogeneous among the included trials. ^b^ Low number of events. ^c^ Extremely low number of events. ^d^ A wide confidence interval, with low number of participants.

## Data Availability

No new data were created or analyzed in this study.

## Supplementary Materials

The following supporting information can be downloaded at: https://www.mdpi.com/article/10.3390/medicina61122103/s1, Table S1: Search Strategy; Table S2: Excluded records in full-text screening.
