# Supplementary material for: Efficacy and Safety of 6.3 Fr Versus 7.5 Fr Single-Use Flexible Ureteroscopes for Upper Urinary Tract Stones: A Systematic Review and Meta-Analysis of Randomized Controlled Trials
Source: Medicina (Kaunas). 2025 Nov 26;61(12):2103. doi: 10.3390/medicina61122103 (PMC12734828; doi:10.3390/medicina61122103)
Supplement: Supplementary file 1 [file medicina-61-02103-s001.zip › medicina-3993862-supplementary.pdf]

Supplementary Materials:

Contents:

Tables.

Table S1: Search strategy.
Table S2: Excluded records in full-text screening.

Table S1: Search Strategy.

**Database**	**Search Terms**	**Search Field**	**Search Results**
PubMed	(ureteroscopy OR ureteroscop* OR RIRS OR "retrograde intrarenal surgery" OR "flexible ureteroscope*") AND ("6.3 Fr" OR "6.3Fr” OR "7.5 Fr" OR "7.5Fr" OR "ultra-slim" OR "ultraslim" OR "small diameter")	All Fields	107
Cochrane	(ureteroscopy OR ureteroscop* OR RIRS OR "retrograde intrarenal surgery" OR "flexible ureteroscope*") AND ("6.3 Fr" OR "6.3Fr” OR "7.5 Fr" OR "7.5Fr" OR "ultra-slim" OR "ultraslim" OR "small diameter")	All Text	37
WOS	(ureteroscopy OR ureteroscop* OR RIRS OR "retrograde intrarenal surgery" OR "flexible ureteroscope*") AND ("6.3 Fr" OR "6.3Fr” OR "7.5 Fr" OR "7.5Fr" OR "ultra-slim" OR "ultraslim" OR "small diameter")	All Fields	106
SCOPUS	TITLE-ABS-KEY ((ureteroscopy OR ureteroscop* OR RIRS OR "retrograde intrarenal surgery" OR "flexible ureteroscope*") AND ("6.3 Fr" OR "6.3Fr" OR "7.5 Fr" OR "7.5Fr" OR "ultra-slim" OR "ultraslim" OR "small diameter"))	Title, Abstract, Keywords	130

Table S2: Excluded records in full-text screening.

Title	Published Year	DOI	Study ID	Exclusion Reason
Two Types of Single-use Flexible Ureteroscopies for the Treatment of Upper Urinary Tract Stones in Children	2024	NCT06628765 2024	NCT06628765 2024	Pediatric population
Efficacy and safety of two tip flexible suctioning ureteral access sheaths combined with a 7.5Fr flexible ureteroscope: a retrospective study.	2025	10.3389/fsurg.2025.1628264	Luo 2025	Wrong study design
In vitro suction comparison of 3 flexible and navigable suction ureteral access sheaths (FANS) of 2 sizes using a 7.5Fr single-use flexible ureteroscope: an AUSET-EAU endourology collaboration.	2025	10.1007/s00345-025-05812-0	Law 2025	Wrong study design
First clinical application of the novel 6.3 Fr disposable digital flexible ureteroscope for treatment of lower calyx renal stones in anticoagulated patient.	2025	10.1016/j.eucr.2025.103015	Xu 2025	Wrong study design
A comparison of deflection, irrigation and optical characteristics of a novel 6.3fr flexible ureteroscope to three contemporary flexible ureteroscopes	2025	10.1097/01.JU.0001109904.25944.a0.14	Crew 2025	Wrong setting
